# 3D cell nuclei segmentation based on gradient flow tracking

**DOI:** 10.1186/1471-2121-8-40

**Published:** 2007-09-04

**Authors:** Gang Li, Tianming Liu, Ashley Tarokh, Jingxin Nie, Lei Guo, Andrew Mara, Scott Holley, Stephen TC Wong

**Affiliations:** 1 Center for Bioinformatics, Harvard Center for Neurodegeneration and Repair, Harvard Medical School, Boston, MA, USA; 2 School of Automation, Northwestern Polytechnic University, Xi'an, China; 3 Functional and Molecular Imaging Center, Department of Radiology, Brigham and Women's Hospital, Boston, MA, USA; 4 Department of Molecular, Cellular and Developmental Biology, Yale University, New Haven, CT, USA

## Abstract

**Background:**

Reliable segmentation of cell nuclei from three dimensional (3D) microscopic images is an important task in many biological studies. We present a novel, fully automated method for the segmentation of cell nuclei from 3D microscopic images. It was designed specifically to segment nuclei in images where the nuclei are closely juxtaposed or touching each other. The segmentation approach has three stages: 1) a gradient diffusion procedure, 2) gradient flow tracking and grouping, and 3) local adaptive thresholding.

**Results:**

Both qualitative and quantitative results on synthesized and original 3D images are provided to demonstrate the performance and generality of the proposed method. Both the over-segmentation and under-segmentation percentages of the proposed method are around 5%. The volume overlap, compared to expert manual segmentation, is consistently over 90%.

**Conclusion:**

The proposed algorithm is able to segment closely juxtaposed or touching cell nuclei obtained from 3D microscopy imaging with reasonable accuracy.

## Background

Reliable segmentation of cell nuclei from three dimensional (3D) microscopic images is an important task in many biological studies as it is required for any subsequent comparison or classification of the nuclei. For example, zebrafish somitogenesis is governed by a clock that generates oscillations in gene expression within the presomitic mesoderm [1,2]. The subcellular localization of oscillating mRNA in each nucleus, imaged through multi-channel microscopy, can be used to identify different phases within the oscillation. To automate the classification of the phase of an individual nucleus, each nucleus within the presomitic mesoderm first needs to be accurately segmented.

In recent years, there has been significant effort towards the development of automated methods for 3D cell or cell nuclei image segmentation [3-9,15,16]. Thresholding, watershed and active surface based methods are among the most commonly used techniques for 3D cell or cell nuclei segmentation. Unfortunately, thresholding-based methods often have difficulties in dealing with images that do not have a well-defined constant contrast between the objects and the background. Given this characteristic of the thresholding-based methods, they often have difficulties in segmenting images with clustered or juxtaposed nuclei. Watershed-based methods are also very popular for segmentation of clustered cell nuclei [3-5,10]. However, these methods often result in the over-segmentation of clustered cell nuclei. In order to deal with this issue, heuristic rules have been developed for region merging [3-5] as a post-processing step. Segmentation problems have also been targeted through the use of active surface-based methods [8,9,15,16] in the literature. However, such algorithms suffer from an inherent dependency on the initial guess. If the initial guess is wrong, these methods have difficulties in dealing with clustered cell nuclei.

Despite active research and progress in the literature, development of a fully automated and robust computational algorithm for 3D cell nuclei segmentation still remains a challenge when dealing with significant inherent nuclei shape and size variations in image data. Examples include cases where the contrast between nuclei and background is low, where there are differences in shapes and sizes of nuclei, and where we are dealing with 3D images of low quality [3,4,6-8]. Complications also arise when nuclei are juxtaposed or connected to one another, increasing the rate of over-segmentation or under-segmentation.

In this paper, we present a novel automated method that aims to tackle the aforementioned challenges of segmentation of clustered or connected 3D cell nuclei. We approach the segmentation problem by first generating the gradient vector field corresponding to the 3D volume image, and then diffusing the gradient vector field with an elastic deformable transform. After the elastic deformable transform is completed, the noisy gradient vector field is smoothed and the gradient vectors with large magnitude are propagated to the areas with weak gradient vectors. This gradient diffusion procedure results in a gradient flow field, in which the gradient vectors are smoothly flowing towards or outwards from the centers of the nuclei. Subsequently, a gradient flow tracking procedure is performed from each vector point to find the corresponding center to which the points flow. We group all points that flow to the same center into a region, and refer to this region as the attraction basin of the center. Once we have completed the process of tracking the gradient flow, the boundaries of juxtaposed nuclei are formed naturally and hence these juxtaposed nuclei are divided. The final step includes performing local thresholding in each attraction basin in order to extract the nuclei from their corresponding background. We have evaluated and validated this algorithm and have presented results attesting its validity.

## Results

In this section, a series of experiments are designed to evaluate and validate the gradient flow tracking method for segmentation of 3D images with juxtaposed nuclei. Both qualitative and quantitative results on synthesized and original 3D images are provided to demonstrate the performance and general applicability of the proposed method.

### Validation using synthesized 3D image

**Figure 5:**
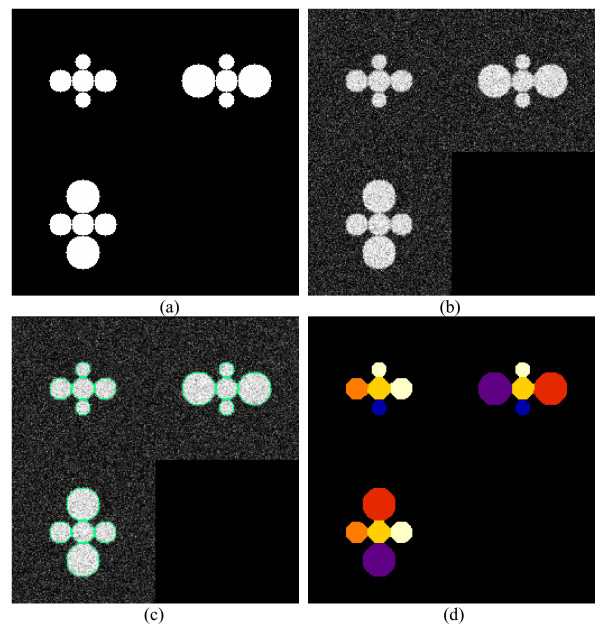
**Result on synthesized 3D image segmentation**. (a) The cross-sectional views of binary mask of the synthesized image. (b) The cross-sectional views of synthesized image with added noise. (c) The cross-sectional views of the overlaid boundaries of segmentation on synthesized noisy 3D image. (d) The cross-sectional views of randomly color-coded segmentation result.

We present an example of the results obtained from applying our proposed nuclei segmentation method on a synthesized image. Despite the fact that objects are touching each other and the presence of additive noise, the proposed segmentation method has segmented the touching objects perfectly, as shown in Figure 5.

We employ volume overlap measurement methods in order to quantitatively validate our segmentation results. Since there is no ground truth information available for real data and it is very time consuming to manually segment juxtaposed nuclei, instead of using real data, we take advantage of synthesized 3D images. The volume overlap between the automated results and the ground truth is defined as:

O(Ra,Rg)=S(Ra∩Rg)(S(Ra)+S(Rg))/2,
 MathType@MTEF@5@5@+=feaafiart1ev1aaatCvAUfKttLearuWrP9MDH5MBPbIqV92AaeXatLxBI9gBaebbnrfifHhDYfgasaacH8akY=wiFfYdH8Gipec8Eeeu0xXdbba9frFj0=OqFfea0dXdd9vqai=hGuQ8kuc9pgc9s8qqaq=dirpe0xb9q8qiLsFr0=vr0=vr0dc8meaabaqaciaacaGaaeqabaqabeGadaaakeaacqWGpbWtcqGGOaakcqWGsbGudaWgaaWcbaGaemyyaegabeaakiabcYcaSiabdkfasnaaBaaaleaacqWGNbWzaeqaaOGaeiykaKIaeyypa0ZaaSaaaeaacqWGtbWucqGGOaakcqWGsbGudaWgaaWcbaGaemyyaegabeaakiablMIijjabdkfasnaaBaaaleaacqWGNbWzaeqaaOGaeiykaKcabaGaeiikaGIaem4uamLaeiikaGIaemOuai1aaSbaaSqaaiabdggaHbqabaGccqGGPaqkcqGHRaWkcqWGtbWucqGGOaakcqWGsbGudaWgaaWcbaGaem4zaCgabeaakiabcMcaPiabcMcaPiabc+caViabikdaYaaacqGGSaalaaa@50C7@

where *R*_*a *_is the automated extracted region and *R*_*g *_is the ground truth region. The ⋂ operator takes the intersection of two regions. *S*(·) is the volume of the region.

**Figure 6:**
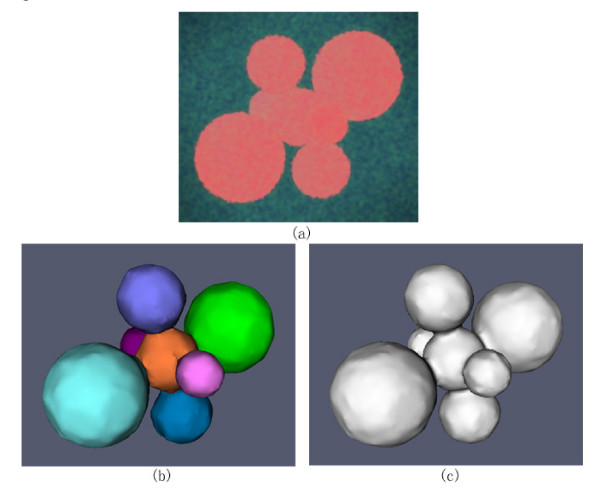
**Volume and surface rendering of synthesized 3D cell nuclei image and segmentation results**. (a) Volume rendering of the synthesized noisy image. (b) Surface rendering of the segmentation results with the proposed method, in which each color indicates a segmented object. (c) Surface rendering of the segmentation results with the global Otsu thresholding, in which the touching objects are not divided correctly.

The synthesized 3D touching cell nuclei image is generated as follows: 1) Randomly select a voxel as seed point, and construct a mask of a sphere with a radius 10 mm centered at that point. 2) Generate 6 masks of spheres, which are tangent to the central sphere, with their corresponding radii ranging from 7 mm to 15 mm. This is done to simulate the variations between radii of real nuclei. 3) Blur the mask images by convolving with a 3D Gaussian kernel, and corrupting it with additive Gaussian noise. For the convenience of visual inspection of the segmentation results, we provide the volume rendering of the original 3D image and surface rendering of the segmentation results. Figure 6a shows the synthesized 3D cell nuclei image, in which the six nuclei are closely touching the central nucleus. The segmentation results with the proposed method are illustrated in Figure 6b. Figure 6c shows the segmenation results obtained by global Otsu thresholding method. As we can see, the touching objects are not divided correctly in figure 6c.

We have synthesized seven 3D nuclei images. In order to present a quantitative measure for the validation of our results, the average value of volume overlap measurement for the seven cases is around 0.971, and the standard deviation is 0.014. As it is clear from these results, our proposed segmentation method achieves significant volume overlap with the ground truth, indicating the accurate performance of the gradient flow tracking method.

### Validation using 3D *C. elegans *embryo images

**Figure 7:**
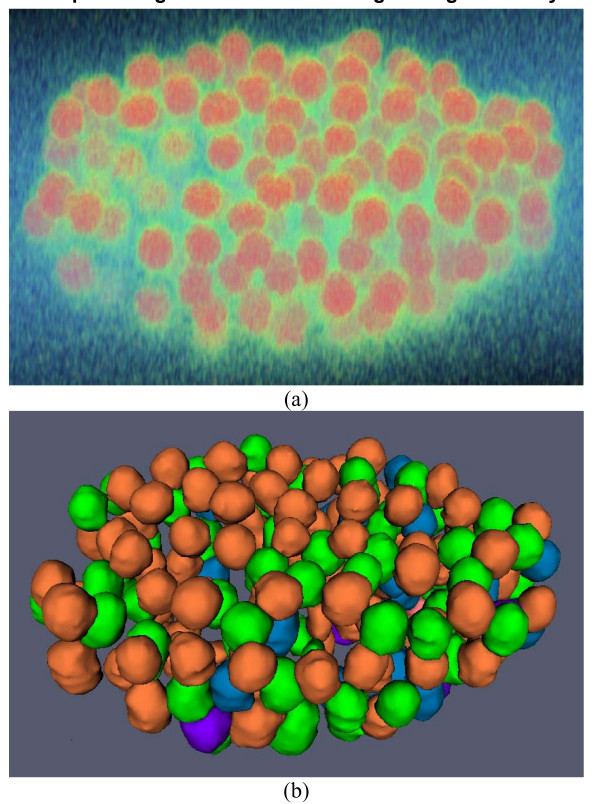
**An example of segmentation result using C. elegans embryo image**. (a) Volume rendering of original 3D image. (b) Surface rendering of segmentation results. For the convenience of inspection of touching cell nuclei, the results are randomly color-coded.

In order to further quantitatively evaluate the segmentation method, we applied the method to *C. elegans *embryo images. Dr. Sean Megason of California Institute of Technology provided us the *C. elegans *embryo images. The nuclei are labelled with histone-EGFP. The scope used for the C. elegans image set is a Zeiss LSM510 with a 40X C-Apochromat objective and a 488 nm laser. The original voxel size is 0.14*0.14*0.74 micron in x, y and z directions. In our experiment, the voxel size is re-sampled to isotropic in all directions. The details of the experimental and imaging settings are provided at . Two metrics of over-segmentation and under-segmentation were utilized for evaluation of the segmentation method. The over-segmentation metric indicates that a nucleus has been separated into more than one object, or an extracted object has not been labeled as nucleus. This is done in comparison to visual inspection of an expert. The under-segmentation indicates that clusters of nuclei have not been appropriately divided or a nucleus marked by visual inspection was not at all extracted. Four 3D images of *C. elegans *embryos were used to evaluate the proposed segmentation method based on the above two metrics. Table 1 shows the performance of the proposed segmentation method. On average, the over-segmentation and under-segmentation rates are 1.59% and 0.39% respectively, indicating a desirable performance by our segmentation method. The errors are probably caused by the interpolation and re-sampling and inherent noise in the images. For the convenience of visual inspection of the segmentation results, we provide the volume rendering of the original 3D image and surface rendering of the segmentation results. As an example, Figure 7a provides the volume rendering of the original juxtaposed 3D nuclei. The segmentation results represented by surface boundaries are shown in Figure 7b. To validate the segmentation results, two experts manually segmented the nuclei respectively, and then we computed the volume overlap between the automated result and that of each of the two experts. We also calculated the volume overlap between the two experts. The mean value of volume overlap is over 95% for both expert 1 and expert 2, and the standard deviation is around 0.02 for both experts, indicating that the automated 3D cell nuclei segmentation results are comparable to manual segmentation results.

**Table 1:** The segmentation results of 3D C. elegans embryo images

**Image Index**	**True Number**	**Over-segmentation**		**Under-segmentation**	
		
		**Number**	**Percentage**	**Number**	**Percentage**
1	187	2	1.07%	0	0.00%
2	187	3	1.60%	1	0.53%
3	185	3	1.62%	0	0.00%
4	192	4	2.08%	2	1.04%
Average	187.75	3	1.59%	0.75	0.39%

### Validation using 3D zebrafish nuclei images

**Figure 8:**
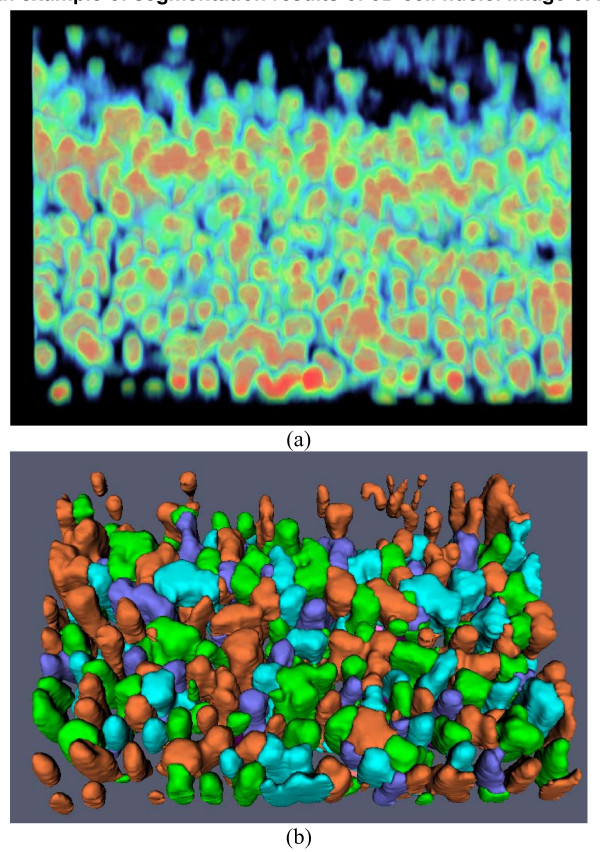
**An example of segmentation results of 3D cell nuclei image of zebrafish**. (a) Volume rendering of original 3D image. (b) Surface rendering of segmentation results. For the convenience of inspection of touching cell nuclei, the results are randomly color-coded.

**Figure 9:**
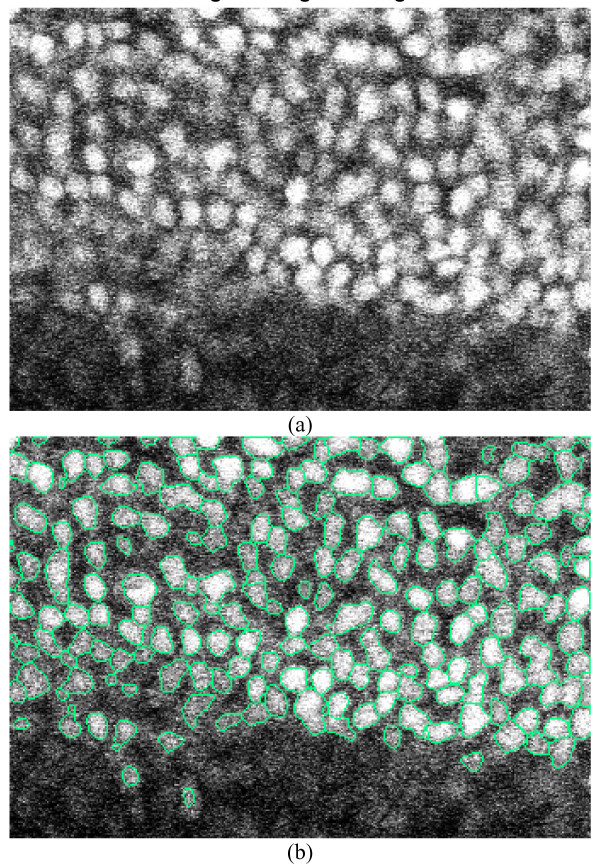
**2D slice view of the original image and segmentation result in Figure 8**. (a). Original image. (b). The segmented image in which the green curves represent the boundaries of the cell nuclei.

To further evaluate the proposed segmentation method, we have applied the method to ten 3D zebrafish images in which nuclei are labeled. The 3D zebrafish image datasets are from the Holley Lab at Yale University. These are fluorescent confocal images of fixed 12 somite stage zebrafish embryos stained with propidium iodide. All images were collected on a BioRad 1024 confocal microscope using a 25X Zeiss Neofluor objective. For the convenience of visual inspection of the segmentation results, we provide the volume rendering of the original 3D image and surface rendering of the segmentation results. An example of the original 3D image and nuclei segmentation results are shown in Figure 8, in which it is evident that most of the nuclei are segmented correctly, in spite of the fact that many of the nuclei are touching and have irregular shapes. Figure 9 shows a 2D slice of the original image and the segmentation result as shown in Figure 8. The two metrics of over-segmentation and under-segmentation are used to evaluate the segmentation result. Table 2 provides the details of the performance of the segmentation method. On average, there exist 310 cell nuclei for each image, and the over-segmentation and under-segmentation rates are 4.93% and 5.03% respectively. Furthermore, the average volume overlap is over 90% for both expert 1 and expert 2, and the standard deviation is less than 0.02 for both experts, indicating a desirable performance by our segmentation method

**Table 2:** The segmentation results of 3D cell nuclei image of zebrafish

**Image Index**	**True Number**	**Over-segmentation**		**Under-segmentation**	
		
		**Number**	**Percentage**	**Number**	**Percentage**
1	283	13	4.59%	15	5.30%
2	309	15	4.85%	16	5.48%
3	320	17	5.31%	15	4.39%
4	293	14	4.78%	13	4.44%
5	325	17	5.23%	19	5.85%
6	306	15	4.90%	17	5.56%
7	332	13	3.92%	19	5.72%
8	304	16	5.26%	15	4.93%
9	311	16	5.14%	13	4.18%
10	320	17	5.31%	17	5.31%
Average	310.3	15.3	4.93%	15.6	5.03%

## Discussion

Our method has several advantages over previous approaches. The major advantage of the method is the ability to robustly segment densely packed, touching, or connected nuclei. Additionally, no sophisticated rules are used. The only assumption is that the centers of nuclei are brighter or darker than a nearby region. The fundamental difference between our method and existing methods lies in the diffused gradient vector information. In existing methods such as the threshold or watershed methods, intensity is the only adopted information, hence those methods are sensitive to the noise in the image, which usually results in over-segmentation. In contrast, in our method the gradient vector diffusion procedure propagates gradient vectors with large magnitudes to the areas with weak gradient vectors and smoothes the noisy gradient vector field. Meanwhile, it preserves the potential structural information of the gradient vector field. For example when two nuclei are touching each other, the diffused gradient vectors point toward the corresponding centers of the nuclei. This step greatly contributes to the success of touching nuclei segmentation. The disadvantage of this method is that it may have difficulty in processing the images of textured blob objects, since in that situation the gradient vector at the centers of nuclei are cluttered and the condition is violated. Currently the method is implemented using the C/C++ language, without using any other common library. Without any optimization, it takes less than 50 seconds on an Intel Pentium4 2.4 GHz machine with 1 GB memory to segment a volume image with a size of 230*162*80. The running time can be reduced further with multi-resolution implementation and code optimisation. After evaluating this method on larger and more diverse image datasets, we intend to release the algorithm to the cell biology community.

## Conclusion

We presented a novel, automated algorithm for 3D cell nuclei segmentation based on gradient flow tracking. To validate the efficacy and performance of the proposed segmentation algorithm, we evaluated it by using synthesized and real biological images. The results show that the algorithm is able to segment juxtaposed nuclei correctly, a persistent problem in the field of cellular image analysis.

## Methods

### Gradient vector diffusion by elastic deformation transformation

Gradient information is an important factor in three-dimensional (3D) nuclei segmentation due to the fact that in any given nuclei image, the gradient vectors either point towards the central area of a bright nucleus, or outwards from the central area of a dark nucleus. However, in practice, the gradient magnitude is very small, and the direction of the gradient vector is usually not trustworthy due to the noise present in the image when approaching the central area of a nucleus. Additionally, when we are dealing with nuclei that are of irregular shapes, the gradient vectors tend to be cluttered. Motivated by these facts, here, we introduce a physical model that incorporates the diffused gradient vectors from the boundaries of the image to generate a smooth gradient field. Our gradient vector diffusion procedure propagates gradient vectors with large magnitudes to the areas with weak gradient vectors and smoothes the noisy gradient vector field [11]. For a detailed introduction to gradient vector diffusion, we refer to [11]. We adopt an elastic deformation transformation, under which the image is modeled as elastic sheets warped by an external force field to achieve gradient vector diffusion. This model has been previously employed for image registration [12,13], where the deformation of boundary points are fixed and then the deformation field is propagated to inner regions of the image by solving the elastic model equation. Here, we extend this model to analyze 3D microscopic nuclei images.

The diffused gradient vector field **v**(*x*, *y*, *z*) = (*u*(*x*, *y*, *z*), *v*(*x*, *y*, *z*), *w*(*x*, *y*, *z*)) (*u*(*x*, *y*, *z*), *v*(*x*, *y*, *z*) and *w*(*x*, *y*, *z*) are three components of the diffused gradient vector projecting to *x*, *y *and *z *axis respectively) in a 3D image is defined to be a solution to the partial differential equation (PDE), also known as a Navier-Stokes equation, describing the deformation of an elastic sheet [13]:

*μ*∇^2^**v **+ (*λ *+ *μ*)∇*div*(**v**) + *q *× (∇*f *- **v**) = 0,

where ∇^2 ^is the Laplacian operator, *div *is the divergence operator, ∇ is the gradient operator, ∇*f *is the original gradient vector field, and Lame's coefficients *μ *and *λ *refer to the elastic properties of the material. In this paper, we aim to diffuse the gradient vectors toward the central areas of nuclei objects to obtain a gradient flow field. Therefore, *f *is set to be

*f *(*x*, *y*, *z*) = *G*_σ _(*x*, *y*, *z*)*I(*x*, *y*, *z*),

where *I*(*x*, *y*, *z*) is a 3D intensity image and *G*_*σ*_(*x*, *y*, *z*) is a 3D Gaussian function with standard derivation *σ*. Note that before computing the convolution and gradient vector, the images should have been interpolated using a spline-based method and re-sampled to isotropic voxel sizes. *q *is a function indicating whether or not the displacement is pre-fixed at the position. In our method, the indicator function is set as

q(x,y,z)={1|∇f(x,y,z)|>Threshold0otherwise
 MathType@MTEF@5@5@+=feaafiart1ev1aaatCvAUfKttLearuWrP9MDH5MBPbIqV92AaeXatLxBI9gBaebbnrfifHhDYfgasaacH8akY=wiFfYdH8Gipec8Eeeu0xXdbba9frFj0=OqFfea0dXdd9vqai=hGuQ8kuc9pgc9s8qqaq=dirpe0xb9q8qiLsFr0=vr0=vr0dc8meaabaqaciaacaGaaeqabaqabeGadaaakeaacqWGXbqCcqGGOaakcqWG4baEcqGGSaalcqWG5bqEcqGGSaalcqWG6bGEcqGGPaqkcqGH9aqpdaGabeqaauaabaqaciaaaeaacqaIXaqmaeaadaabdaqaaiabgEGirlabdAgaMjabcIcaOiabdIha4jabcYcaSiabdMha5jabcYcaSiabdQha6jabcMcaPaGaay5bSlaawIa7aiabg6da+iabdsfaujabdIgaOjabdkhaYjabdwgaLjabdohaZjabdIgaOjabd+gaVjabdYgaSjabdsgaKbqaaiabicdaWaqaaiabb+gaVjabbsha0jabbIgaOjabbwgaLjabbkhaYjabbEha3jabbMgaPjabbohaZjabbwgaLbaaaiaawUhaaaaa@618E@

In our current implementation, the *Threshold *is set to be 0. Once the *Threshold *is large, the gradient vectors with small magnitudes will be omitted, including some noisy gradient vectors and some useful gradient vectors. Therefore, the *Threshold *creates a compromise between keeping useful gradient vectors and removing noisy gradient vectors. The model is solved by treating *u*, *v *and *w *as functions of time:

{vt(x,y,z,t)=μ∇2v(x,y,z,t)+(λ+μ)∇div(v(x,y,z,t))+q(x,y,z)(∇f(x,y,z)−v(x,y,z,t))v(x,y,z,0)=∇f(x,y,z)
 MathType@MTEF@5@5@+=feaafiart1ev1aaatCvAUfKttLearuWrP9MDH5MBPbIqV92AaeXatLxBI9gBaebbnrfifHhDYfgasaacH8akY=wiFfYdH8Gipec8Eeeu0xXdbba9frFj0=OqFfea0dXdd9vqai=hGuQ8kuc9pgc9s8qqaq=dirpe0xb9q8qiLsFr0=vr0=vr0dc8meaabaqaciaacaGaaeqabaqabeGadaaakeaadaGabeqaauaabaqaceaaaeaacqWH2bGDdaWgaaWcbaGaemiDaqhabeaakiabcIcaOiabdIha4jabcYcaSiabdMha5jabcYcaSiabdQha6jabcYcaSiabdsha0jabcMcaPiabg2da9GGaciab=X7aTjabgEGirpaaCaaaleqabaGaeGOmaidaaOGaeCODayNaeiikaGIaemiEaGNaeiilaWIaemyEaKNaeiilaWIaemOEaONaeiilaWIaemiDaqNaeiykaKIaey4kaSIaeiikaGIae83UdWMaey4kaSIae8hVd0MaeiykaKIaey4bIeTaemizaqMaemyAaKMaemODayNaeiikaGIaeCODayNaeiikaGIaemiEaGNaeiilaWIaemyEaKNaeiilaWIaemOEaONaeiilaWIaemiDaqNaeiykaKIaeiykaKIaey4kaSIaemyCaeNaeiikaGIaemiEaGNaeiilaWIaemyEaKNaeiilaWIaemOEaONaeiykaKIaeiikaGIaey4bIeTaemOzayMaeiikaGIaemiEaGNaeiilaWIaemyEaKNaeiilaWIaemOEaONaeiykaKIaeyOeI0IaeCODayNaeiikaGIaemiEaGNaeiilaWIaemyEaKNaeiilaWIaemOEaONaeiilaWIaemiDaqNaeiykaKIaeiykaKcabaGaeCODayNaeiikaGIaemiEaGNaeiilaWIaemyEaKNaeiilaWIaemOEaONaeiilaWIaeGimaaJaeiykaKIaeyypa0Jaey4bIeTaemOzayMaeiikaGIaemiEaGNaeiilaWIaemyEaKNaeiilaWIaemOEaONaeiykaKcaaaGaay5Eaaaaaa@A04A@

where **v**_*t*_(*x*, *y*, *z*, *t*) denotes the partial derivative of **v**(*x*, *y*, *z*, *t*) with respect to time *t*. The equation is decoupled as:

*u*_*t*_(*x*, *y*, *z*, *t*) = *μ*∇^2^*u*(*x*, *y*, *z*, *t*) + (*λ *+ *μ*) (∇*div*(**v**(*x*, *y*, *z*, *t*)))_*x *_+ *q*(*x*, *y*, *z*)((∇*f*(*x*, *y*, *z*))_*x *_- *u*(*x*, *y*, *z*, *t*))

*v*_*t *_(*x*, *y*, *z*, *t*) = *μ*∇^2^*v*(*x*, *y*, *z*, *t*) + (*λ *+ *μ*) (∇*div*(**v**(*x*, *y*, *z*, *t*)))_*y *_+ *q*(*x*, *y*, *z*)((∇*f*(*x*, *y*, *z*))_*y *_- *v*(*x*, *y*, *z*, *t*))

*w*_*t*_(*x*, *y*, *z*, *t*) = *μ*∇^2^*w*(*x*, *y*, *z*, *t*) + (*λ *+ *μ*) (∇*div*(**v**(*x*, *y*, *z*, *t*)))_*z *_+ *q*(*x*, *y*, *z*)((∇*f*(*x*, *y*, *z*))_*z *_- *w*(*x*, *y*, *z*, *t*))

With the finite difference method, by setting the spacing interval Δ*x*, Δ*y*, Δ*z *and time interval Δ*t *all to be 1 and letting the indices *i*, *j*, *k *and *n *correspond to *x*, *y*, *z *and *t *respectively, the equations are approximated as:

ut=ui,j,kn+1−ui,j,kn,vt=vi,j,kn+1−vi,j,kn,wt=wi,j,kn+1−wi,j,kn,∇2u=ui+1,j,k+ui−1,j,k+ui,j+1,k+ui,j−1,k+ui,j,k+1+ui,j,k−1−6ui,j,k∇2v=vi+1,j,k+vi−1,j,k+vi,j+1,k+vi,j−1,k+vi,j,k+1+vi,j,k−1−6vi,j,k∇2w=wi+1,j,k+wi−1,j,k+wi,j+1,k+wi,j−1,k+wi,j,k+1+wi,j,k−1−6wi,j,k(∇div(v))x=ui+1,j,k+ui−1,j,k−2ui,j,k+vi+1,j+1,k−vi,j+1,k−vi+1,j,k+vi,j,k+wi+1,j,k+1−wi,j,k+1−wi+1,j,k+wi,j,k(∇div(v))y=vi,j+1,k+vi,j−1,k−2vi,j,k+ui+1,j+1,k−ui+1,j,k−ui,j+1,k+ui,j,k+wi,j+1,k+1−wi,j,k+1−wi,j+1,k+wi,j,k(∇div(v))z=wi,j,k+1+wi,j,k−1−2wi,j,k+ui+1,j,k+1−ui+1,j,k−ui,j,k+1+ui,j,k+vi,j+1,k+1−vi,j,k+1−vi,j+1,k+vi,j,k(∇f)x=fi+1,j,k−fi,j,k,(∇f)y=fi,j+1,k−fi,j,k,(∇f)z=fi,j,k+1−fi,j,k
 MathType@MTEF@5@5@+=feaafiart1ev1aaatCvAUfKttLearuWrP9MDH5MBPbIqV92AaeXatLxBI9gBaebbnrfifHhDYfgasaacH8akY=wiFfYdH8Gipec8Eeeu0xXdbba9frFj0=OqFfea0dXdd9vqai=hGuQ8kuc9pgc9s8qqaq=dirpe0xb9q8qiLsFr0=vr0=vr0dc8meaabaqaciaacaGaaeqabaqabeGadaaakeaafaqaaeWcbaaaaaqaauaabeqabmaaaeaacqWG1bqDdaWgaaWcbaGaemiDaqhabeaakiabg2da9iabdwha1naaDaaaleaacqWGPbqAcqGGSaalcqWGQbGAcqGGSaalcqWGRbWAaeaacqWGUbGBcqGHRaWkcqaIXaqmaaGccqGHsislcqWG1bqDdaqhaaWcbaGaemyAaKMaeiilaWIaemOAaOMaeiilaWIaem4AaSgabaGaemOBa4gaaOGaeiilaWcabaGaemODay3aaSbaaSqaaiabdsha0bqabaGccqGH9aqpcqWG2bGDdaqhaaWcbaGaemyAaKMaeiilaWIaemOAaOMaeiilaWIaem4AaSgabaGaemOBa4Maey4kaSIaeGymaedaaOGaeyOeI0IaemODay3aa0baaSqaaiabdMgaPjabcYcaSiabdQgaQjabcYcaSiabdUgaRbqaaiabd6gaUbaakiabcYcaSaqaaiabdEha3naaBaaaleaacqWG0baDaeqaaOGaeyypa0Jaem4DaC3aa0baaSqaaiabdMgaPjabcYcaSiabdQgaQjabcYcaSiabdUgaRbqaaiabd6gaUjabgUcaRiabigdaXaaakiabgkHiTiabdEha3naaDaaaleaacqWGPbqAcqGGSaalcqWGQbGAcqGGSaalcqWGRbWAaeaacqWGUbGBaaGccqGGSaalaaaabaGaey4bIe9aaWbaaSqabeaacqaIYaGmaaGccqWG1bqDcqGH9aqpcqWG1bqDdaWgaaWcbaGaemyAaKMaey4kaSIaeGymaeJaeiilaWIaemOAaOMaeiilaWIaem4AaSgabeaakiabgUcaRiabdwha1naaBaaaleaacqWGPbqAcqGHsislcqaIXaqmcqGGSaalcqWGQbGAcqGGSaalcqWGRbWAaeqaaOGaey4kaSIaemyDau3aaSbaaSqaaiabdMgaPjabcYcaSiabdQgaQjabgUcaRiabigdaXiabcYcaSiabdUgaRbqabaGccqGHRaWkcqWG1bqDdaWgaaWcbaGaemyAaKMaeiilaWIaemOAaOMaeyOeI0IaeGymaeJaeiilaWIaem4AaSgabeaakiabgUcaRiabdwha1naaBaaaleaacqWGPbqAcqGGSaalcqWGQbGAcqGGSaalcqWGRbWAcqGHRaWkcqaIXaqmaeqaaOGaey4kaSIaemyDau3aaSbaaSqaaiabdMgaPjabcYcaSiabdQgaQjabcYcaSiabdUgaRjabgkHiTiabigdaXaqabaGccqGHsislcqaI2aGncqWG1bqDdaWgaaWcbaGaemyAaKMaeiilaWIaemOAaOMaeiilaWIaem4AaSgabeaaaOqaaiabgEGirpaaCaaaleqabaGaeGOmaidaaOGaemODayNaeyypa0JaemODay3aaSbaaSqaaiabdMgaPjabgUcaRiabigdaXiabcYcaSiabdQgaQjabcYcaSiabdUgaRbqabaGccqGHRaWkcqWG2bGDdaWgaaWcbaGaemyAaKMaeyOeI0IaeGymaeJaeiilaWIaemOAaOMaeiilaWIaem4AaSgabeaakiabgUcaRiabdAha2naaBaaaleaacqWGPbqAcqGGSaalcqWGQbGAcqGHRaWkcqaIXaqmcqGGSaalcqWGRbWAaeqaaOGaey4kaSIaemODay3aaSbaaSqaaiabdMgaPjabcYcaSiabdQgaQjabgkHiTiabigdaXiabcYcaSiabdUgaRbqabaGccqGHRaWkcqWG2bGDdaWgaaWcbaGaemyAaKMaeiilaWIaemOAaOMaeiilaWIaem4AaSMaey4kaSIaeGymaedabeaakiabgUcaRiabdAha2naaBaaaleaacqWGPbqAcqGGSaalcqWGQbGAcqGGSaalcqWGRbWAcqGHsislcqaIXaqmaeqaaOGaeyOeI0IaeGOnayJaemODay3aaSbaaSqaaiabdMgaPjabcYcaSiabdQgaQjabcYcaSiabdUgaRbqabaaakeaacqGHhis0daahaaWcbeqaaiabikdaYaaakiabdEha3jabg2da9iabdEha3naaBaaaleaacqWGPbqAcqGHRaWkcqaIXaqmcqGGSaalcqWGQbGAcqGGSaalcqWGRbWAaeqaaOGaey4kaSIaem4DaC3aaSbaaSqaaiabdMgaPjabgkHiTiabigdaXiabcYcaSiabdQgaQjabcYcaSiabdUgaRbqabaGccqGHRaWkcqWG3bWDdaWgaaWcbaGaemyAaKMaeiilaWIaemOAaOMaey4kaSIaeGymaeJaeiilaWIaem4AaSgabeaakiabgUcaRiabdEha3naaBaaaleaacqWGPbqAcqGGSaalcqWGQbGAcqGHsislcqaIXaqmcqGGSaalcqWGRbWAaeqaaOGaey4kaSIaem4DaC3aaSbaaSqaaiabdMgaPjabcYcaSiabdQgaQjabcYcaSiabdUgaRjabgUcaRiabigdaXaqabaGccqGHRaWkcqWG3bWDdaWgaaWcbaGaemyAaKMaeiilaWIaemOAaOMaeiilaWIaem4AaSMaeyOeI0IaeGymaedabeaakiabgkHiTiabiAda2iabdEha3naaBaaaleaacqWGPbqAcqGGSaalcqWGQbGAcqGGSaalcqWGRbWAaeqaaaGcbaGaeiikaGIaey4bIeTaemizaqMaemyAaKMaemODayNaeiikaGIaeCODayNaeiykaKIaeiykaKYaaSbaaSqaaiabdIha4bqabaGccqGH9aqpcqWG1bqDdaWgaaWcbaGaemyAaKMaey4kaSIaeGymaeJaeiilaWIaemOAaOMaeiilaWIaem4AaSgabeaakiabgUcaRiabdwha1naaBaaaleaacqWGPbqAcqGHsislcqaIXaqmcqGGSaalcqWGQbGAcqGGSaalcqWGRbWAaeqaaOGaeyOeI0IaeGOmaiJaemyDau3aaSbaaSqaaiabdMgaPjabcYcaSiabdQgaQjabcYcaSiabdUgaRbqabaGccqGHRaWkcqWG2bGDdaWgaaWcbaGaemyAaKMaey4kaSIaeGymaeJaeiilaWIaemOAaOMaey4kaSIaeGymaeJaeiilaWIaem4AaSgabeaakiabgkHiTiabdAha2naaBaaaleaacqWGPbqAcqGGSaalcqWGQbGAcqGHRaWkcqaIXaqmcqGGSaalcqWGRbWAaeqaaOGaeyOeI0IaemODay3aaSbaaSqaaiabdMgaPjabgUcaRiabigdaXiabcYcaSiabdQgaQjabcYcaSiabdUgaRbqabaGccqGHRaWkcqWG2bGDdaWgaaWcbaGaemyAaKMaeiilaWIaemOAaOMaeiilaWIaem4AaSgabeaaaOqaaiabgUcaRiabdEha3naaBaaaleaacqWGPbqAcqGHRaWkcqaIXaqmcqGGSaalcqWGQbGAcqGGSaalcqWGRbWAcqGHRaWkcqaIXaqmaeqaaOGaeyOeI0Iaem4DaC3aaSbaaSqaaiabdMgaPjabcYcaSiabdQgaQjabcYcaSiabdUgaRjabgUcaRiabigdaXaqabaGccqGHsislcqWG3bWDdaWgaaWcbaGaemyAaKMaey4kaSIaeGymaeJaeiilaWIaemOAaOMaeiilaWIaem4AaSgabeaakiabgUcaRiabdEha3naaBaaaleaacqWGPbqAcqGGSaalcqWGQbGAcqGGSaalcqWGRbWAaeqaaaGcbaGaeiikaGIaey4bIeTaemizaqMaemyAaKMaemODayNaeiikaGIaeCODayNaeiykaKIaeiykaKYaaSbaaSqaaiabdMha5bqabaGccqGH9aqpcqWG2bGDdaWgaaWcbaGaemyAaKMaeiilaWIaemOAaOMaey4kaSIaeGymaeJaeiilaWIaem4AaSgabeaakiabgUcaRiabdAha2naaBaaaleaacqWGPbqAcqGGSaalcqWGQbGAcqGHsislcqaIXaqmcqGGSaalcqWGRbWAaeqaaOGaeyOeI0IaeGOmaiJaemODay3aaSbaaSqaaiabdMgaPjabcYcaSiabdQgaQjabcYcaSiabdUgaRbqabaGccqGHRaWkcqWG1bqDdaWgaaWcbaGaemyAaKMaey4kaSIaeGymaeJaeiilaWIaemOAaOMaey4kaSIaeGymaeJaeiilaWIaem4AaSgabeaakiabgkHiTiabdwha1naaBaaaleaacqWGPbqAcqGHRaWkcqaIXaqmcqGGSaalcqWGQbGAcqGGSaalcqWGRbWAaeqaaOGaeyOeI0IaemyDau3aaSbaaSqaaiabdMgaPjabcYcaSiabdQgaQjabgUcaRiabigdaXiabcYcaSiabdUgaRbqabaGccqGHRaWkcqWG1bqDdaWgaaWcbaGaemyAaKMaeiilaWIaemOAaOMaeiilaWIaem4AaSgabeaaaOqaaiabgUcaRiabdEha3naaBaaaleaacqWGPbqAcqGGSaalcqWGQbGAcqGHRaWkcqaIXaqmcqGGSaalcqWGRbWAcqGHRaWkcqaIXaqmaeqaaOGaeyOeI0Iaem4DaC3aaSbaaSqaaiabdMgaPjabcYcaSiabdQgaQjabcYcaSiabdUgaRjabgUcaRiabigdaXaqabaGccqGHsislcqWG3bWDdaWgaaWcbaGaemyAaKMaeiilaWIaemOAaOMaey4kaSIaeGymaeJaeiilaWIaem4AaSgabeaakiabgUcaRiabdEha3naaBaaaleaacqWGPbqAcqGGSaalcqWGQbGAcqGGSaalcqWGRbWAaeqaaaGcbaGaeiikaGIaey4bIeTaemizaqMaemyAaKMaemODayNaeiikaGIaeCODayNaeiykaKIaeiykaKYaaSbaaSqaaiabdQha6bqabaGccqGH9aqpcqWG3bWDdaWgaaWcbaGaemyAaKMaeiilaWIaemOAaOMaeiilaWIaem4AaSMaey4kaSIaeGymaedabeaakiabgUcaRiabdEha3naaBaaaleaacqWGPbqAcqGGSaalcqWGQbGAcqGGSaalcqWGRbWAcqGHsislcqaIXaqmaeqaaOGaeyOeI0IaeGOmaiJaem4DaC3aaSbaaSqaaiabdMgaPjabcYcaSiabdQgaQjabcYcaSiabdUgaRbqabaGccqGHRaWkcqWG1bqDdaWgaaWcbaGaemyAaKMaey4kaSIaeGymaeJaeiilaWIaemOAaOMaeiilaWIaem4AaSMaey4kaSIaeGymaedabeaakiabgkHiTiabdwha1naaBaaaleaacqWGPbqAcqGHRaWkcqaIXaqmcqGGSaalcqWGQbGAcqGGSaalcqWGRbWAaeqaaOGaeyOeI0IaemyDau3aaSbaaSqaaiabdMgaPjabcYcaSiabdQgaQjabcYcaSiabdUgaRjabgUcaRiabigdaXaqabaGccqGHRaWkcqWG1bqDdaWgaaWcbaGaemyAaKMaeiilaWIaemOAaOMaeiilaWIaem4AaSgabeaaaOqaaiabgUcaRiabdAha2naaBaaaleaacqWGPbqAcqGGSaalcqWGQbGAcqGHRaWkcqaIXaqmcqGGSaalcqWGRbWAcqGHRaWkcqaIXaqmaeqaaOGaeyOeI0IaemODay3aaSbaaSqaaiabdMgaPjabcYcaSiabdQgaQjabcYcaSiabdUgaRjabgUcaRiabigdaXaqabaGccqGHsislcqWG2bGDdaWgaaWcbaGaemyAaKMaeiilaWIaemOAaOMaey4kaSIaeGymaeJaeiilaWIaem4AaSgabeaakiabgUcaRiabdAha2naaBaaaleaacqWGPbqAcqGGSaalcqWGQbGAcqGGSaalcqWGRbWAaeqaaaGcbaqbaeqabeWaaaqaaiabcIcaOiabgEGirlabdAgaMjabcMcaPmaaBaaaleaacqWG4baEaeqaaOGaeyypa0JaemOzay2aaSbaaSqaaiabdMgaPjabgUcaRiabigdaXiabcYcaSiabdQgaQjabcYcaSiabdUgaRbqabaGccqGHsislcqWGMbGzdaWgaaWcbaGaemyAaKMaeiilaWIaemOAaOMaeiilaWIaem4AaSgabeaakiabcYcaSaqaaiabcIcaOiabgEGirlabdAgaMjabcMcaPmaaBaaaleaacqWG5bqEaeqaaOGaeyypa0JaemOzay2aaSbaaSqaaiabdMgaPjabcYcaSiabdQgaQjabgUcaRiabigdaXiabcYcaSiabdUgaRbqabaGccqGHsislcqWGMbGzdaWgaaWcbaGaemyAaKMaeiilaWIaemOAaOMaeiilaWIaem4AaSgabeaakiabcYcaSaqaaiabcIcaOiabgEGirlabdAgaMjabcMcaPmaaBaaaleaacqWG6bGEaeqaaOGaeyypa0JaemOzay2aaSbaaSqaaiabdMgaPjabcYcaSiabdQgaQjabcYcaSiabdUgaRjabgUcaRiabigdaXaqabaGccqGHsislcqWGMbGzdaWgaaWcbaGaemyAaKMaeiilaWIaemOAaOMaeiilaWIaem4AaSgabeaaaaaaaaaa@1B0F@

**Figure 1:**
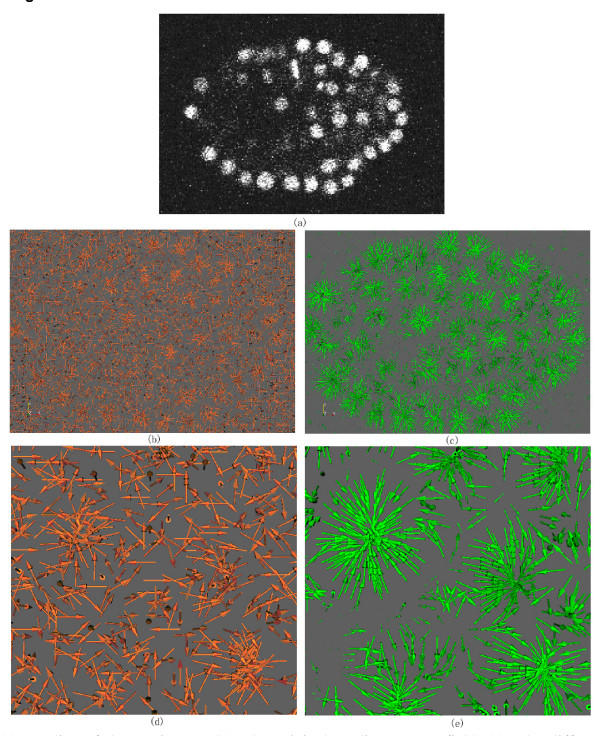
**The 3D view of gradient vector field and diffused gradient vector field with elastic deformation transformation of a slice cropped from a 3D cell nuclei image**. (a). A slice of the 3D image. (b). The original gradient vector field. (c). The diffused gradient flow field with elastic deformation transformation. Obviously, the diffused vector field with elastic deformation transformation smoothly flows toward the central areas of cell nuclei. (d). Zoomed view of (b). (e). Zoomed view of (c).

The solution to Equation 2 defines the displacement of each position in a 3D elastic object, where displacements at some locations are pre-fixed. In Equation 2, variable **v **represents velocity and hence, considering hydromechanics rules, the second term in Equation 2 denotes the compression level of a compressible fluid. Given this description, setting *div*(**v**) = 0 represents an uncompressible fluid. The terms *μ *and *λ *in Equation 2 determine the tradeoff between conformability to the pre-fixed deformation vectors and smoothness of the deformation field [13]. As it is clear from Equation 2, when *μ *and *λ *are small, the pre-fixed deformation vectors are preserved. Moreover, having large values for terms *μ *and *λ *will result in obtaining a smoother deformation field. As an example, in Figure 1, we demonstrate a comparison between the zoomed diffused gradient vector field with elastic deformation transformation and the original gradient vector field of a slice from a 3D nucleus image. As is clear from Figure 1, the diffused vector field using the elastic deformable model flows more smoothly towards the central areas of nuclei compared to the original gradient vector field. Moreover, even though nuclei are closely juxtaposed, the diffused flow field splits along a clear boundary and flows towards the corresponding central areas of each nucleus. This property greatly contributes to the success of 3D nucleus segmentation.

### Gradient flow tracking

In the diffused gradient vector field, the vectors flow toward the sinks, which correspond to the centers of nuclei. To follow the vectors until they stop at the sinks, the gradient flow tracking procedure is performed as follows. From any starting point **x **= (*x*, *y*, *z*), the next point **x**' = (*x'*, *y'*, *z'*) that **x **flows through in the diffused gradient field is computed as:

x′=x+round(v(x)‖v(x)‖)
 MathType@MTEF@5@5@+=feaafiart1ev1aaatCvAUfKttLearuWrP9MDH5MBPbIqV92AaeXatLxBI9gBaebbnrfifHhDYfgasaacH8akY=wiFfYdH8Gipec8Eeeu0xXdbba9frFj0=OqFfea0dXdd9vqai=hGuQ8kuc9pgc9s8qqaq=dirpe0xb9q8qiLsFr0=vr0=vr0dc8meaabaqaciaacaGaaeqabaqabeGadaaakeaacuWH4baEgaqbaiabg2da9iabhIha4jabgUcaRiabdkhaYjabd+gaVjabdwha1jabd6gaUjabdsgaKnaabmaabaWaaSaaaeaacqWH2bGDcqGGOaakcqWH4baEcqGGPaqkaeaadaqbdaqaaiabhAha2jabcIcaOiabhIha4jabcMcaPaGaayzcSlaawQa7aaaaaiaawIcacaGLPaaaaaa@46A7@

Here, **v**(**x**) is the diffused gradient vector at point **x**, and "round" returns the nearest integer. The angle between the diffused gradient vectors of these two adjacent points is determined as:

θ=arccos⁡〈v(x)‖v(x)‖,v(x′)‖v(x′)‖〉
 MathType@MTEF@5@5@+=feaafiart1ev1aaatCvAUfKttLearuWrP9MDH5MBPbIqV92AaeXatLxBI9gBaebbnrfifHhDYfgasaacH8akY=wiFfYdH8Gipec8Eeeu0xXdbba9frFj0=OqFfea0dXdd9vqai=hGuQ8kuc9pgc9s8qqaq=dirpe0xb9q8qiLsFr0=vr0=vr0dc8meaabaqaciaacaGaaeqabaqabeGadaaakeaaiiGacqWF4oqCcqGH9aqpcyGGHbqycqGGYbGCcqGGJbWycqGGJbWycqGGVbWBcqGGZbWCdaaadaqaamaalaaabaGaeCODayNaeiikaGIaeCiEaGNaeiykaKcabaWaauWaaeaacqWH2bGDcqGGOaakcqWH4baEcqGGPaqkaiaawMa7caGLkWoaaaGaeiilaWYaaSaaaeaacqWH2bGDcqGGOaakcuWH4baEgaqbaiabcMcaPaqaamaafmaabaGaeCODayNaeiikaGIafCiEaGNbauaacqGGPaqkaiaawMa7caGLkWoaaaaacaGLPmIaayPkJaaaaa@536D@

**Figure 2:**
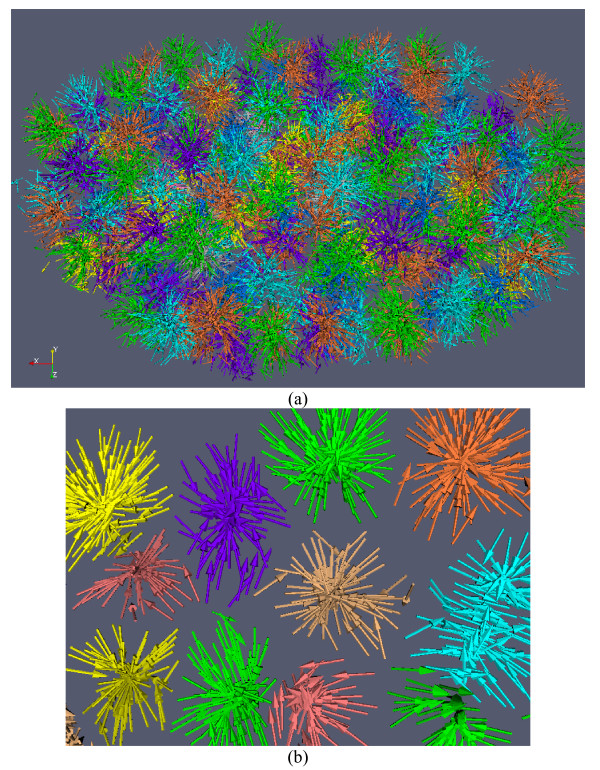
**Illustration of the gradient flow tracking based segmentation method**. (a). Clustered gradient vectors in 3D space. (b). Zoomed view of selected clusters of gradient vectors. Each color represents a separated cell.

When the angle between two consecutive diffused gradient vectors is less than 90 degrees, the gradient flow tracking procedure continues. Otherwise, the gradient flow tracking procedure is stopped, and a sink is reached. In this way, the vectors at each point along the tracking curve define a smooth path leading to a sink. In practice, segmentation of images into nuclei can be obtained by starting a gradient flow tracking procedure from every point in image. The set of pixels that flow to the same sink naturally produce the attraction basin of the sink. All points in the same attraction basin are segmented as an object (nucleus). All points are tracked independently, thus the attraction basin can be of arbitrary shape. After the gradient flow tracking step if the sinks are very close to each other, the attraction basins of the sink are combined together to obtain a larger attraction basin. In all our experiments, if the distance between two sinks is less than three pixels the attraction basins of the two sinks are combined together to obtain a larger attraction basin. Figure 2 shows an illustration of the result of the gradient flow tracking based method.

The algorithm of the gradient flow tracking is summarized as follows.

1. Randomly select a point **x **as the initial point **x**^0^.

2. Obtain **x**^*n *+ 1 ^(*n *= 0,1,2...) using Equation 3 based on **x**^*n*^.

3. Compute the angle *θ*_*n *_of diffused gradient vector between **x**^*n *+ 1 ^and **x**^*n *^with Equation 4. If *θ*_*n *_is larger than π2
 MathType@MTEF@5@5@+=feaafiart1ev1aaatCvAUfKttLearuWrP9MDH5MBPbIqV92AaeXatLxBI9gBaebbnrfifHhDYfgasaacH8akY=wiFfYdH8Gipec8Eeeu0xXdbba9frFj0=OqFfea0dXdd9vqai=hGuQ8kuc9pgc9s8qqaq=dirpe0xb9q8qiLsFr0=vr0=vr0dc8meaabaqaciaacaGaaeqabaqabeGadaaakeaadaWcaaqaaGGaciab=b8aWbqaaiabikdaYaaaaaa@2F72@, stop.

4. Replace **x**^*n *^with **x**^*n *+ 1^. Return to step 2.

Gradient flow tracking is applied to each point in the image. All points in the same attraction basin are grouped into the same cluster. Since it is time consuming to run the tracking algorithm for every point, in order to improve the performance of our method, gradient flow tracking is not applied to the points that have already been on the gradient flow trajectory of a previously processed pixel. Instead, these visited points are directly associated with the sink to which the path flows. This improvement not only speeds up the segmentation, but also yields reproducible segmentation results.

### Local adaptive thresholding

After the gradient flow tracking step, the image is segmented into smaller regions each of which is expected to contain only a single nucleus. From here the nuclei segmentation problem is turned into binary classification problem where we are interested in distinguishing the nuclei from their background in a small region. Therefore an intensity thresholding method is appropriate for extracting the nuclei from the background. In order to approach this problem, we can take advantage of the method employed by Otsu in [14], which has the ability to extract the nucleus from each attraction basin. Another approach for dealing with this problem is through designing a more involved method that employs techniques such as graph cut, level set, etc. Here, we employ the locally adaptive method of Otsu [14] because of its ability to deal with situations where the intensity of nuclei and background are not constant across an image. In each segmented region, pixels whose intensities are larger than the automatically determined local Otsu threshold are grouped as nuclei, otherwise they are grouped as background. Finally, an optional procedure is performed after extracting the nuclei to eliminate small regions, which contain a lower number of pixels than a threshold.

### Summary of 3D cell nuclei segmentation method

The algorithm of the 3D cell nuclei segmentation method based on gradient flow tracking is summarized as follows.

1. Obtain the diffuse gradient vector field using the elastic deformation transformation

2. From each point, run the gradient flow tracking procedure, and label each passed pixel with a converged sink position.

3. Combine the attraction basins of the sinks whose distance is less than three pixels.

4. Assign the same label to the points in the same attraction basin.

5. Perform local adaptive thresholding in each attraction basin to extract the nucleus.

6. Optional procedure: eliminate regions with smaller number of pixels than a threshold T.

### Running example

**Figure 3:**
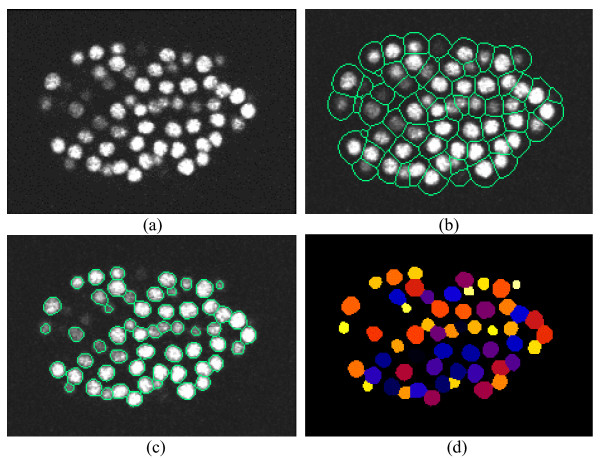
**Illustration of 3D cell nuclei segmentation on a 2D slice**. (a) A slice from 3D cell nuclei image. (b)Boundaries of small regions overlaid on the slice. The boundaries are obtained by the method in Section 2.2. (c) Edges of cell nuclei overlaid on the slice after the step of adaptive thresholding in Section 2.3. (d) Randomly color-coded extracted cells.

Figure 3 provides an illustration of 3D nuclei segmentation on a 2D slice. In Figure 3b, we demonstrate the initial subdivision of the image into nuclei areas after the gradient flow tracking procedure. As it is clear from these images, each nucleus is enclosed by a boundary. The nuclei segmentation results after the adaptive thresholding is provided in Figure 3c, and their randomly color-coded representation is shown in Figure 3d.

**Figure 4:**
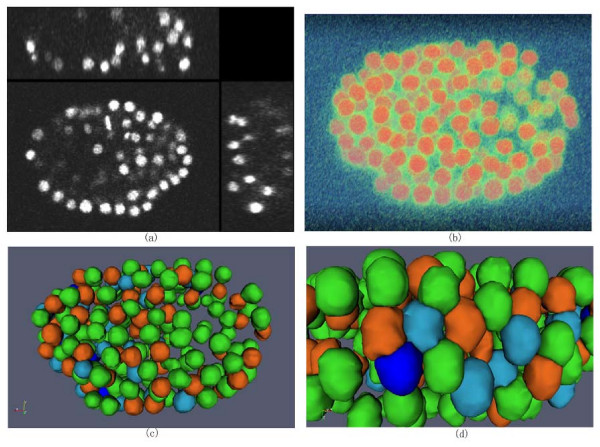
**Illustration of cell nuclei segmentation in 3D space**. (a). Cross-sectional views of a 3D cell nuclei image. (b) Volume rendering of 3D cell nuclei image. (c) Surface rendering of extracted 3D cell nuclei. For the convenience of inspection of touching cell nuclei, the results are randomly color-coded. (d) Zooming view of the results.

Figure 4 provides an illustration of the 3D nuclei segmentation procedure in the 3D space. In Figure 4a, the cross-sectional views of a 3D image are shown. Figure 4b renders the boundary surfaces of extracted 3D nuclei. For the convenience of inspection of touching nuclei, the extracted nuclei are randomly color-coded.

## Authors' contributions

GL and TL proposed the algorithm. GL implemented the algorithm. JN helped implement the algorithm. AT and STCW verified the algorithm and the result. AM and SH acquired the image data and provided biological input to this work.

## Supplementary Material

Additional file 1 The computer program, called CellSegmentation3D, loads the 3D Analyze format image (the suffix ".img" or ".hdr" is not needed in the input image name), and the segmentation result is also saved as the Analyze format. For each 3D input image, the program will output two results: 1) the segmentation result, in which all voxels belonging to the same cell are labelled with the same unique intensity; 2) the boundary map that separates segmented cells. Usage: CellSegmentation3D image_Input -f fusion_threshold -m min_Region -d diffusion_iteration -s sigma; Default parameters: fusion_threshold 3, min_Region 50, diffusion_iteration 15, sigma 1.0; For example: CellSegmentation3D elegans-01-01 -f 3 -m 35. This will produce the two result images: *elegans-01-01_edge.img *and *elegans-01-01_segmentation.img*. Notes that the segmentation results can be inspected by many visualization tools, provided that they can load 3D Analyze format images. The NIH ImageJ progam (freely downloadable at: ) is recommended. Click here for file

Additional file 2 The elegans-01-01.img is an example image file. Click here for file

Additional file 3 The elegans-01-01.hdr is the header file associated with the image. Click here for file
